# Effect of 160 MeV Xenon Ion Irradiation on the Tribological Properties and Crystal Structure of 100Cr6 Bearing Steel

**DOI:** 10.3390/ma16206660

**Published:** 2023-10-12

**Authors:** Mariusz Kamiński, Piotr Budzyński, Zbigniew Surowiec, Marek Wiertel, Maxim V. Zdorovets, Artem Kozlovskiy, Janusz Waliszewski, Marek Magdziak

**Affiliations:** 1 Faculty of Mechanical Engineering, Lublin University of Technology, Nadbystrzycka 36, 20-618 Lublin, Poland; p.budzynski@pollub.pl; 2 Institute of Physics, Maria Curie-Sklodowska University, Pl. Marii Curie-Skłodowskiej 1, 20-031 Lublin, Poland; zbigniew.surowiec@mail.umcs.pl (Z.S.); marek.wiertel@mail.umcs.pl (M.W.); 3 Lab Solid State Phys, Institute of Nuclear Physics, Ibragimov Str. 1, Almaty 050032, Kazakhstan; mzdorovets@inp.kz (M.V.Z.); kozlovskiy.a@inp.kz (A.K.); 4 Engn Profile Lab, L.N. Gumilyov Eurasian National University, Satpayev Str. 2, Astyana 010008, Kazakhstan; 5 Faculty of Physics, University of Bialystok, K. Ciołkowskiego 1L, 15-245 Bialystok, Poland; j.waliszewski@uwb.edu.pl; 6 Department of Manufacturing Techniques and Automation, Faculty of Mechanical Engineering and Aeronautics, Rzeszów University of Technology, al. Powstańców Warszawy 12, 35-959 Rzeszów, Poland; marekm@prz.edu.pl

**Keywords:** irradiation, xenon ions, bearing steel, tribological properties, crystal lattice parameters

## Abstract

This is the first study ever to show the impact of high-energy 160 MeV xenon ion irradiation on the properties of 100Cr6 bearing steel. The projected range (R_p_) of xenon ions is 8.2 µm. Fluence-dependent variations in the coefficient of friction and wear of the 100Cr6 steel material have been observed. These changes correlate with shifts in the crystal lattice constant and variations in the oxygen, carbon, and iron content in the wear track. Fluence-dependent changes in these parameters have been observed for the first time. Irradiation reduces stresses in the crystal lattice, leading to crystallite size increase. The modifications in the properties of 100Cr6 steel result from radiation-induced defects caused by electronic ion stopping. The degree of these modifications depends on the applied irradiation fluence. Furthermore, the use of a higher irradiation fluence value appears to mitigate the effects produced by a lower fluence.

## 1. Introduction

System components operating in outer space as well as those used in nuclear reactors and devices should exhibit a high degree of reliability, despite being continuously bombarded by high-energy ions (nuclear fission fragments of ^235^U). During irradiation, swift ions penetrate a metallic target and transfer their kinetic energy to this target via inelastic collisions with electrons and via elastic collisions with atomic nuclei of the target. The collisions result in the formation of point defects. During migration, some point defects either undergo annealing (they disappear) or form cascades of secondary defects. This is a long-lasting process that can also take place at room temperature [1,2]. Most studies investigate the annealing and evolution of radiation defects as a function of sample temperature [3]. Simulations of cascade aging with displacement at 290 °C in Fe-based alloys have shown that defect migration and recombination can take up to about 24.5 h. This means that when the next ion arrives, defects formed in the previous cascade continue to migrate and can be affected by the incoming ion. It is generally accepted that the evolution of defects and their annihilation practically stops once the irradiation is completed [2]. However, this process has not yet been experimentally studied. There are few works on the evolution of radiation defects depending on the passage of time after irradiation at a fixed temperature [4].

These secondary defects are known as irradiation defects, and they cause changes in the structural, mechanical, tribological, and electrical properties of the irradiated target. The irradiation process changes the topography of a metal surface. The high energy of a heavy ion beam may cause local melting of the surface layer of metals and phase transformation of the crystal structure. Irradiation can lead to the formation of new phases [5]. The presence of irradiation-induced defects usually leads to increased crystal cell volume, which, in turn, results in increased size (swelling) of products (tools, machine parts). The volume of the irradiated layer can increase even by several percent, which is not recommended in the case of precision mechanical elements. 

The irradiation of nanolayers can also lead to component segregation and local changes in alloy density [6,7,8]. This type of segregation can also occur in steels. Most of the previous research has been conducted for the AISI 316 grade steel, which is most often used as a construction material for devices exposed to high-intensity ionizing radiation. The irradiation of this steel grade by 2 MeV protons [9] and 8 MeV iron ions [10] changes its crystal structure and mechanical properties. An amount of 160 keV iron ion irradiation causes an increase in the grain size [11]. A study [12] investigated the crystal structure and defects induced by 7 MeV Xe^26+^ ion irradiation in cold-worked austenitic stainless steel (CW 316 SS). The widening of the XRD peaks indicated the irradiation-induced changes in the lattice parameters. The lattice constant increased with an irradiation fluence. Titanium and its alloy, Ti6Al4V, irradiated with xenon ions showed changes in their crystal lattice parameters tribological properties [13].

The 100Cr6 grade steel is used for high-load mechanical elements such as movable components of rolling bearings [14]. After quenching and tempering, 100Cr6 exhibits high hardness and uniformity, good wear resistance, and high contact fatigue strength. The chemical composition of this steel grade is as follows: C: 0.95–1.1; Cr: 1.3–1.65; Mn: 0.25–0.45; Si: 0.15–0.35; Ni, Cu: ≤0.30; P, S ≤ 0.025; Fe: balance. 

Intensive research is being conducted worldwide to develop construction materials with mechanical and tribological properties that are highly resistant to long-term neutron irradiation exposure. Ion beam irradiation is used for modeling the effects of irradiation caused by neutrons without their activation, leading to neutron radiation emission [15]. It turns out that radiation defects resulting from several-year-long radiation in a nuclear reactor can be induced by a high-energy ion beam within several hours. This greatly facilitates the development of neutron irradiation-resistant materials suitable for the design of critical nuclear reactor components. On the other hand, the microstructures created at high defect rates can greatly differ from those formed at low rates due to the following differences between neutron and ion irradiation: fluence size, primary atom recoil spectra, and helium production. 

A study [16] reported changes in the crystal lattice constant for a thin (≤0.1 µm) surface layer of 100Cr6 steel irradiated with low-energy (≤1 keV) xenon ions. Many previous studies used xenon ions with an energy of about 160 MeV. In our study, we also used 160 MeV xenon ions in order to compare our results with those reported in previous studies. In engineering practice, 160 MeV xenon ions are used for producing nuclear membranes and for testing the cosmic radiation resistance of integrated circuits. Our study focuses on changes in the tribological properties, surface topography, and crystal structure of 100Cr6 steel irradiated with 160 MeV xenon ions. Our results are unique in terms of showing the effects of high-energy ions on the properties of 100Cr6 bearing steel. The friction coefficient and wear of the 100Cr6 grade steel were investigated. This steel grade can be used for movable parts in nuclear reactors as well as those operating in cosmic space and thus exposed to high-intensity ionizing radiation. This study is the first one ever to report fluctuating changes in the properties of 100Cr6 steel with increasing irradiation fluence. This means that even a high irradiation fluence does not have to cause significant changes in the tribological properties of bearing steel; hence, the irradiated element does not have to be replaced with a new one.

## 2. Materials and Methods 

**Figure 1:**
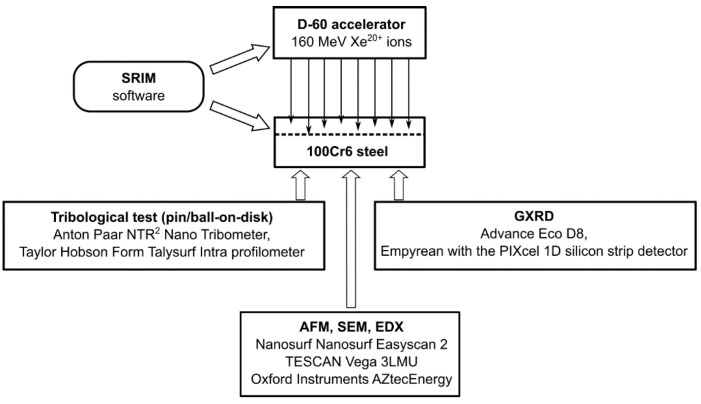
Schematic design of the experimental procedure for testing the properties of 100Cr6 steel irradiated with 160 MeV Xe^20+^ ions.

The test specimens were made from commercial 100Cr6 steel alloy (Variant 803J, C: 1.0%, Si: 0.35%, Mn: 0.40%, P: 0.025%, S: 0.015%, Cr: 1.60%, Ni: 0.25%, Mo: 0.08%), manufactured by Ovako AB, Stockholm, Sweden. The specimens were made from 2-mm-thick, hardened and tempered sheet metal in the form of discs with a diameter of 25 mm, which were cut by waterjet. The samples were then polished to a mirror-like surface. A schematic design of the experiment is shown in Figure 1. The irradiation process was performed in a swift heavy ion accelerator D-60 (L.N. Gumilyov Eurasian National University, Astana, Kazakhstan). To ensure that the uniformity of the irradiation fluence was higher than 5%, the beam was swept horizontally and vertically over the sample surface. The temperature of the sample did not exceed 60 °C during the irradiation process. The pressure in the irradiation chamber was below 3 × 10^−7^ Torr. The ^132^Xe ion beam had an energy of 160 MeV. The samples were irradiated with a fluence of 1 × 10^14^, 2.5 × 10^14^ and 5 × 10^14^ Xe^20+^/cm^2^, respectively.

Changes on the surface of the irradiated samples were examined via atomic force microscopy (AFM) using the Nanosurf Easyscan 2 (Nanosurf AG, Liestal, Switzerland). The samples were measured in air. The microscope was installed on an active anti-vibration table TS-150 (Thorlabs Inc., Newton, NJ, USA). The atomic force microscope cantilevers were calibrated using the Sader method [17]. AFM images of the irradiated layer were processed using the WSXM software (WSxM 5.0 Develop 10.3) [18]. SEM images were captured with a magnification of 600 times. The microscope was calibrated in compliance with the SEM calibration standards for low magnifications in the 5–1000 range. The spectra of characteristic X-rays were measured using the SEM TESCAN Vega 3LMU (Tescan Group, a.s. Brno – Kohoutovice, Czech Republic) coupled with the Oxford Instruments AZtecEnergy—Advanced EDX microanalyzer (Oxford Instruments, High Wycombe, UK). The EDX microanalyzer was calibrated using a set of universal reference standards for X-ray microanalysis (MAC Universal 55 Reference Standard). With this standard, the detectability of elements was at a level of 0.1% by weight, while the uncertainty varied from 0.01 to 0.09% by weight. 

Tribological testing was carried out via the pin/ball-on-disk method using the Anton Paar nanotribometer (NTR^2^) (Anton Paar GmbH, Graz, Austria). The tests were conducted in compliance with the ASTM G99-17 Standard Test Method for Wear Testing with a Pin-on-Disk Apparatus [19]. The counter sample was a silicon carbide (SiC) ball with a diameter of 1.1 mm loaded with a force of 500 mN. The tests were performed under technically dry friction conditions without wear product suction, at a temperature of 22 °C, and with a relative air humidity of 30%. The rotational speed of the sample was 1 rev/s, which corresponded to the sample’s linear velocity of 6.3 cm/s relative to the counter sample. The measurement acquisition frequency was 10 Hz. The test length was set to 10,000 revolutions, and the accumulated sliding distance was about 690 m. The degree of wear was determined using the Taylor Hobson Form Talysurf Intra profilometer (Taylor Hobson Ltd., Leicester, UK) by measuring the cross-sectional area of the wear track made on the sample by the counter sample at 15 points on the wear track perimeter. A measuring needle with a rounding radius of 2 μm was used for the measurements. The measurement range of the measurement head used was 2 mm, while its measurement resolution was 8 nm. After that, sample volume loss (mm^3^) and wear rate were calculated as a ratio of sample volume loss to counter sample loading and friction path (mm^3^/Nm). The wear of the counter sample (ball) was determined by measuring the surface area of the worn part of the ball as expressed in mm^2^, and then its wear rate was measured. The tribometer was calibrated by the manufacturer. The profilometer was calibrated using the roughness standard and contour master provided by the manufacturer.

The crystal structure was examined using two independent X-ray diffractometers: Advance Eco D8 (Bruker, Billerica, MA, USA) in L.N. Gumilyov Eurasian National University and Empyrean in JINR Dubna The Empyrean diffractometer with a PIXcel 1D (Malvern Panalytical Ltd, Malvern, UK) silicon strip detector containing 255 strips and covering a range of 3.5° in 2θ scale was used for data acquisition. This system enables fast measurements with high statistics.

The instrumental integral breadth was determined by measuring a strain-free lanthanum hexaboride (LaB_6_) powder standard (NIST Standard Reference Material 660c) [20] under the same conditions as those applied for the test samples.

## 3. Results and Discussion 

### 3.1. Steel Surface Topography

**Figure 2:**
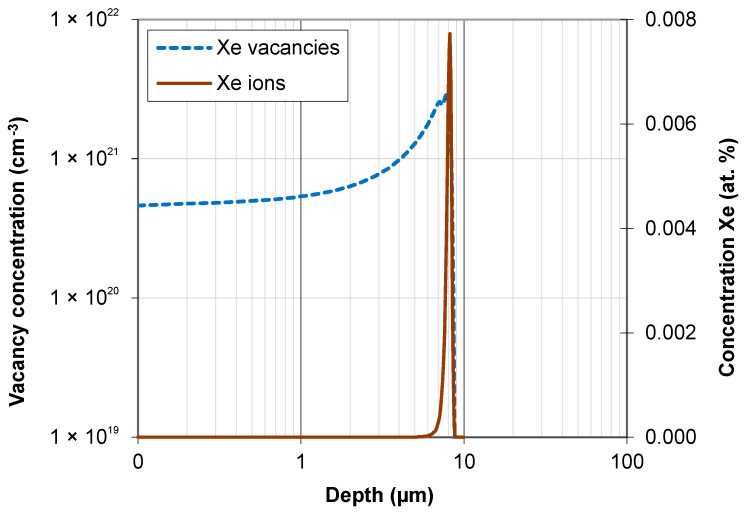
Projected range of 160 MeV Xe ions and Xe vacancies in bearing steel samples.

The irradiation-induced damage profile and injected ion concentration were estimated using SRIM-2013 [21]. All ions are practically stopped in the target at a depth ranging from 6 to 8.2 µm. The projected range R_p_ of xenon ions is 8.2 µm (Figure 2). The vacancy concentration increases with depth. It is about 5 × 10^20^ vac/cm^3^ in the surface layer. The greatest depth of the wear track after tribological testing is 2 µm. For this depth value, the vacancy concentration is 6.35 × 10^21^ cm^−3^. 

**Figure 3:**
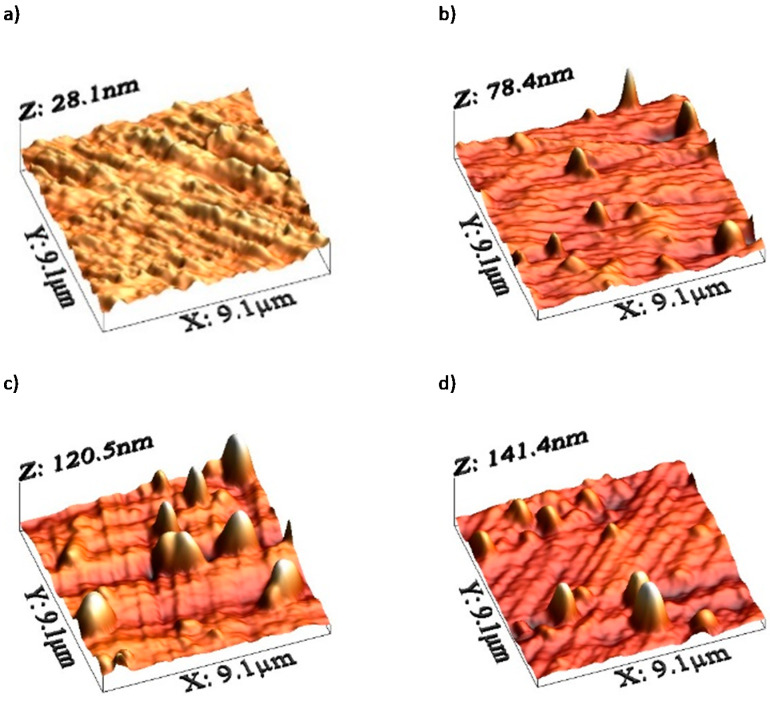
AFM images of the sample surface after primary oxide layer removal: (**a**) unirradiated sample; irradiated with a fluence of (**b**) 1 × 10^14^ Xe^20+^/cm^2^, (**c**) 2.5 × 10^14^ Xe^20+^/cm^2^, (**d**) 5 × 10^14^ Xe^20+^/cm^2^.

AFM images of the steel surface are shown in Figure 3. 

Irradiation causes only small changes in the content of basic steel elements in the surface layer with a thickness below 1 μm (Table 1). As the fluence increases, the content of oxygen increases. Radiation defects make it easier for oxygen to penetrate the sample. The radiation-enhanced diffusion coefficient can exceed the thermal diffusion coefficient by several hundred times [2,22,23]. 

### 3.2. Tribological Measurements 

**Figure 4:**
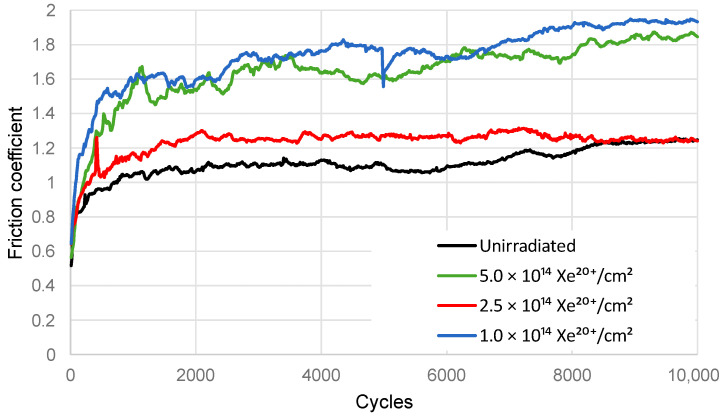
Coefficient of friction of the samples and SiC ball under dry friction conditions.

Results of the coefficient of friction are shown in Figure 4. After irradiation, the friction coefficient of the steel samples increases. The sample irradiated with a fluence of 1 × 10^14^ Xe^20+^/cm^2^ has a significantly higher coefficient of friction compared to the unirradiated sample. On the other hand, the friction coefficient of the sample irradiated with a fluence of 2.5 × 10^14^ Xe^20+^/cm^2^ is lower than that of the previous sample, whereas the fluence of 5 × 10^14^ Xe^20+^/cm^2^ makes the previous friction coefficient value higher and comparable to that obtained for the sample irradiated with a fluence of 1 × 10^14^ Xe^20+^/cm^2^. It can be concluded that an increase in the irradiation fluence causes unusual, irregular changes in the mean coefficient of friction.

**Figure 5:**
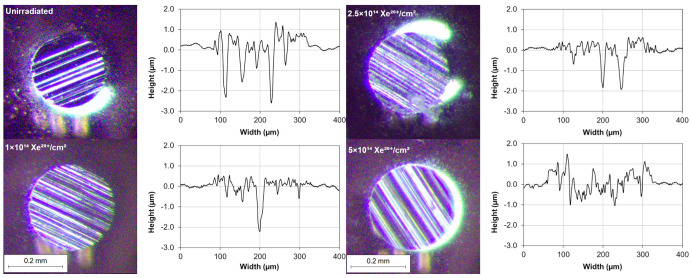
Microscopy images of the worn surface of the SiC counter sample after tribological testing and the wear profile of the bearing steel sample measured with a profilometer.

Profilograms of the wear track made by the SiC counter sample on the steel surface are shown in Figure 5. Instead of one cavity, a series of grooves can be observed, which indicates extensive abrasive wear of both the steel sample and the SiC counter sample (Figure 5).

SEM images of the worn surface of the counter sample as well as the presence of numerous grooves in the wear track on the sample surface indicate that the abrasive wear mechanism is dominant. The friction pair components have similar hardness. Wear debris of the sample remain on its surface and—when pressed against by the SiC ball—cause further wear of the sample. The presence of wear products near the track may be facilitated by a low magnetic impact of the counter sample wear debris. It was shown in [24] that defects in SiC wear debris induced magnetic interactions.

**Figure 6:**
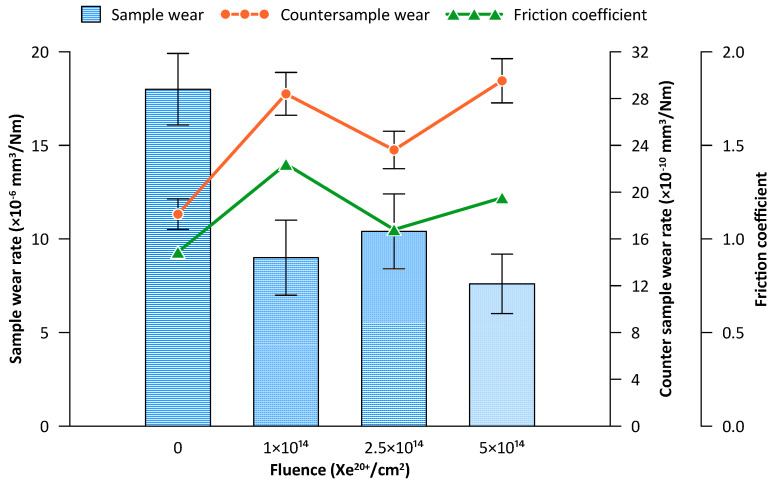
Wear of the bearing steel sample (blue) and counter sample (orange) during the tribological test of 10,000 cycles. The figure also shows the changes in the mean coefficient of friction between 2000 and 6000 tribological test cycles (green).

Wear results for the tribologically tested samples are shown in Figure 6. Irradiation with a fluence of 1 × 10^14^ Xe^20+^/cm^2^ causes an almost two-fold reduction in wear. Although this fluence value is optimal in terms of the degree of wear, it generates the highest friction coefficient value during the tribological test (Figure 6). A further increase in the irradiation fluence causes irregular changes in wear. 

Figure 6 additionally shows changes in the mean coefficient of friction for the range of 2000–6000 tribological test cycles. It can be observed that the wear of the counter sample (Figure 6) is correlated with the changes in the coefficient of friction. It also undergoes fluctuating changes depending on the irradiation fluence. Both the counter sample wear and the coefficient of friction show fluctuating changes as a function of irradiation fluence in the opposite phase to the sample wear changes.

### 3.3. EDX Measurements

**Figure 7:**
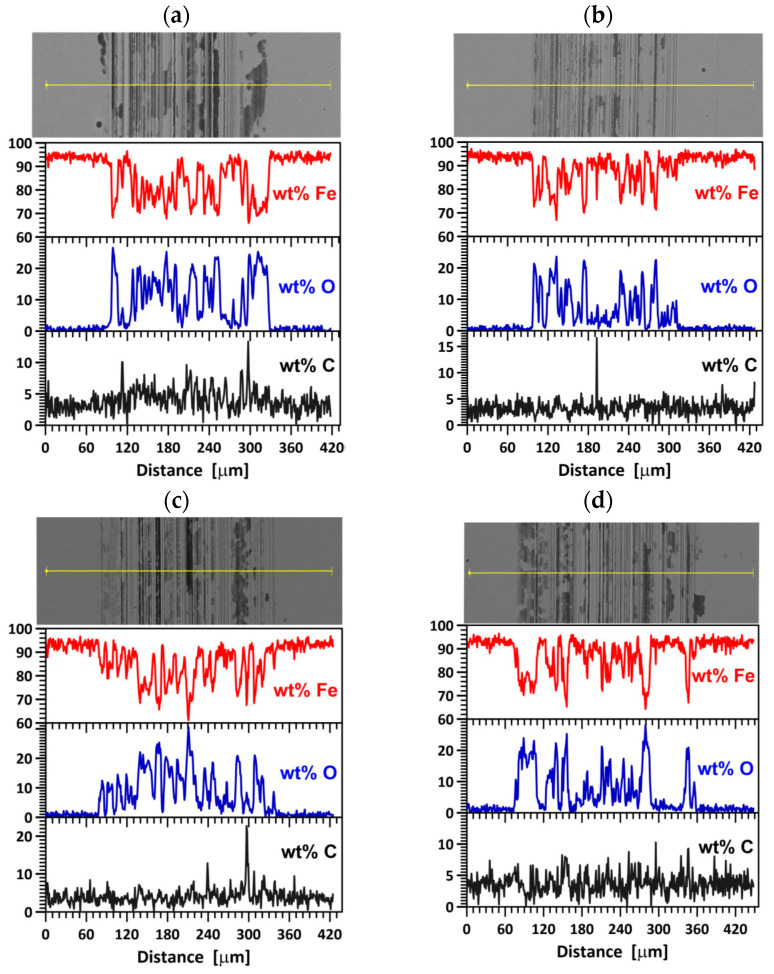
SEM images of the wear track on the sample surface and EDS results of the elements affecting friction: (**a**) unirradiated, (**b**) irradiated with a fluence 1 × 10^14^ Xe^20+^/cm^2^, (**c**) 2.5 × 10^14^ Xe^20+^/cm^2^, (**d**) 5 × 10^14^ Xe^20+^/cm^2^.

Figure 7 shows the SEM images of the wear track on the steel surface as well as the content of iron, oxygen, and carbon along the scan line. The average content of the elements in the wear track on the steel surface after 10,000 tribological test cycles is given in Table 2. Interesting observations can be made regarding the fluctuating changes in the content of the elements that play a decisive role in the friction process, i.e., Fe, C, and O. These fluctuating changes depend on the irradiation fluence. This study is the first one to show the fluctuating changes in the content of these elements in the wear track. The data shown in Figure 7 and in Table 2 indicate abrasive, adhesive, and oxidation wear in the unirradiated sample. In the sample irradiated with a fluence of 1 × 10^14^ Xe^20+^/cm^2^, the abrasive wear mechanism is dominant. When the friction coefficient reaches the maximum value (Figure 4), the wear of the steel sample is the lowest (Figure 6), while that of the counter sample is the highest (Figure 6). A higher xenon ion fluence causes changes in the contribution of abrasive, adhesive, and oxidative wear, which leads to fluctuating changes in the wear of the sample.

### 3.4. GXRD Measurements

The crystal structure of the samples was examined through grazing incidence X-ray diffraction (GXRD). By changing the X-ray beam incidence angle Θ, it is possible to change the thickness of a layer from which data are acquired. The effective penetration depth for each X-ray incidence angle was calculated according to [25], assuming that 63% of the diffracted intensity originated from a volume confined by the depth below the sample surface. The obtained incidence angles and the corresponding calculated penetration depths for CuK_α_ irradiation are listed in Table 3.

**Figure 8:**
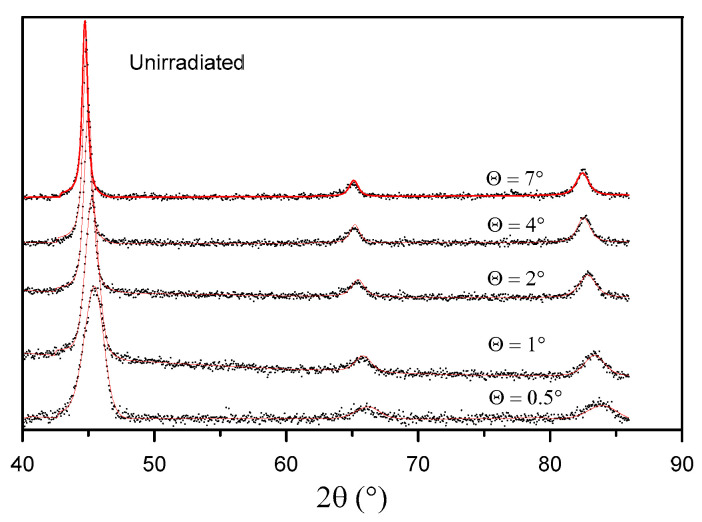
GXRD spectra of the unirradiated sample.

**Figure 9:**
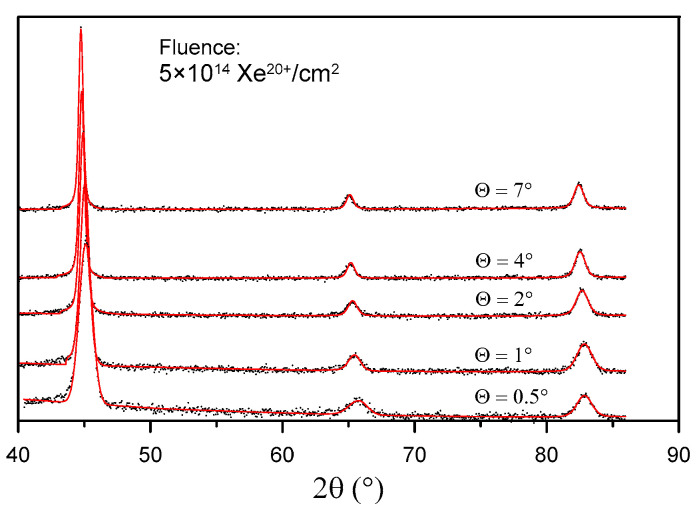
GXRD spectra of the sample irradiated with a fluence of 5 × 10^14^ Xe^20+^/cm^2^.

The GXRD results show the changes in the crystal structure of the sample surface layer at various penetration depths before irradiation (Figure 8) and after irradiation with 160 MeV Xe ions (Figure 9).

**Figure 10:**
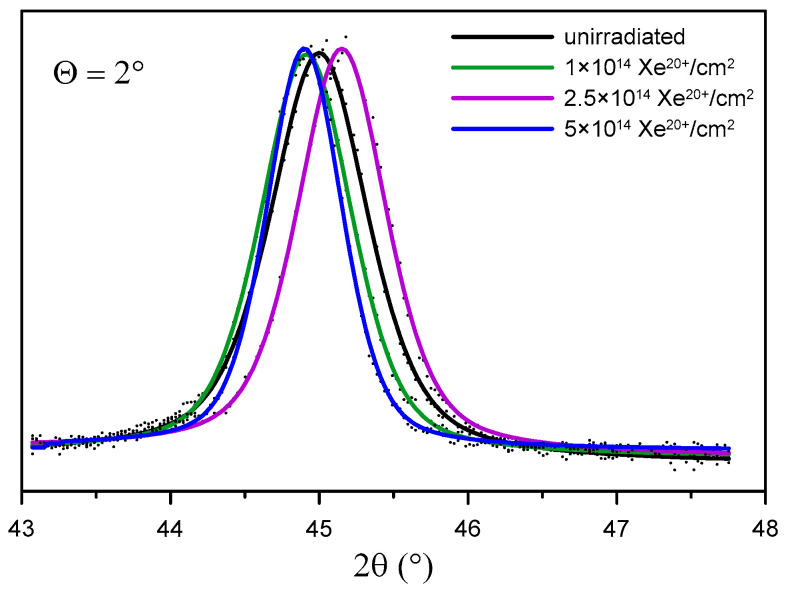
Location of reflection (011) at different depths of sample layers depending on the irradiation fluence.

Figure 10 shows the location of the reflection (011) in GXRD patterns for Θ = 2° obtained for the sample before and after irradiation. It can be observed that this reflection changes its location after irradiation. Unusual (fluctuating) changes in the location of the reflection (011) occur in the same phase for all layers: Θ = 0.5°, 1°,2°, 4°, and 7°. The shift in the location of the reflection point (011) with the change in the irradiation fluence proves that the crystal lattice constant *a* undergoes changes. In Table 4, the values of the crystal lattice constant *a* for the X-ray beam incidence angle Θ = 7° and the change in relation to the value prior to irradiation are listed. Similar changes in the reflection point location for irradiated titanium and its alloy, Ti6A4V, were observed in [13]. The changes can be explained by multi-step damage accumulation in irradiated crystals [26]. The crystal lattice constant usually increases with increasing metal irradiation fluence [27,28]. 

The Bragg peak widths were analyzed using the Williamson–Hall (W-H) method [29]. Briefly, the W-H method allows a rough estimation of the average strain and average crystallite size from the integral width of diffraction peaks. 

Crystal lattice strains and crystallite sizes are given in Table 5. In the unirradiated samples, the greatest crystal lattice strains and the smallest crystallite sizes are located in the surface layer with a thickness of approx. 0.06 µm. As the fluence increases, the crystal lattice strains decrease while the crystallite size increases. In the samples irradiated with a fluence of 5 × 10^14^ Xe^20+^/cm^2^, the parameters *η* and *D* of the surface layer are practically the same as those obtained for the layers located deep inside the sample. As the depth (incidence angle) increases, the crystal lattice strain decreases and the crystallite size increases.

### 3.5. Energy Losses of Xe Ions in the Bearing Steel Target

**Figure 11:**
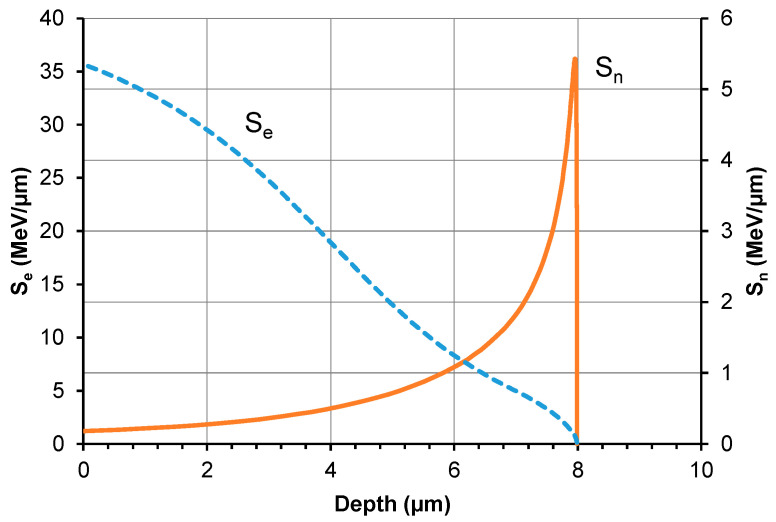
Electronic energy loss (S_e_) and nuclear energy loss (S_n_) of 160 MeV Xe ions versus different depths of the steel target.

The irradiation-induced changes in the tribological properties and crystalline structure of bearing steel are attributed to the energy transfer from swift xenon ions during their deceleration in the bearing steel target. Figure 11 illustrates the theoretical distribution of electronic and nuclear energy losses of 160 MeV xenon ions as computed with SRIM [21]. The wear track obtained in the tribological test is about 2.5 µm deep. From this, it follows that irradiation-induced changes in the mechanical properties of the samples are only caused by electronic energy loss (S_e_) and radiation defects. At this depth value, the impact of xenon ions (S_n_) is insignificant. The defects produced during nuclear stopping are located at a depth ranging from 7 to 9 (µm), with their highest concentration observed at 8.2 µm. The nuclear energy loss in a layer with a thickness of up to 2.5 µm is approx. 0.2 MeV/µm, which is significantly lower than the electron energy loss value ranging from 26 to 36 MeV/µm. 

## 4. Conclusions

Irradiation causes changes in the friction coefficient of steel samples during tribological testing. The mean coefficient of friction changes in a fluctuating manner during the test, depending on the irradiation fluence. The lowest wear can be observed for the samples irradiated with a fluence of 5 × 10^14^ Xe^20+^/cm^2^. The pattern of changes in sample wear with increasing xenon ion fluence fluctuates. The fluctuating changes in the tribological properties of the irradiated bearing steel target are due to surface topography changes (Figure 3), variations in the content of oxygen and carbon—the two elements playing the key role in the friction process (Table 2 and Figure 7)—as well as due to changes in the crystal lattice parameters (Table 4 and Table 5). The surface morphology changes in the irradiated steel target affect the coefficient of friction and the degree of wear during the first 2000 tribological test cycles (Figure 2). The crystallite size and crystal lattice strains affect the degree of wear obtained in the tribological tests. All these changes are caused by radiation defects and energy transfer to steel during electronic and nuclear stopping of xenon ions. 

Following the irradiation with a fluence of 1 × 10^14^ Xe^20+^/cm^2^, the abrasive wear mechanism becomes dominant. When the friction coefficient reaches the maximum value, the wear of the steel sample is minimal while that of the counter sample is maximal. An increase in the xenon ion fluence causes changes in the adhesive and oxidative wear rates, which, in turn, lead to fluence-dependent changes in the wear of the steel samples. The wear of the counter sample also undergoes fluctuating changes with increasing the irradiation fluence.

In addition, irradiation causes fluence-dependent changes in the crystal lattice constant. They result from the annealing of lower fluence-irradiation-induced radiation defects due to the use of a higher fluence (longer irradiation time). Xenon ions transfer their kinetic energy to steel during electronic and nuclear stopping. The electronic energy loss mechanism is dominant in the outer sample layer, which is approx. 2.5 µm thick. Its values range from 26 to 36 MeV per µm. This leads to annealing of radiation defects, reduced crystal lattice strains, and increased crystallite size. 

This study is the first one to observe the fluence-dependent changes for five friction-related parameters: the coefficient of friction, wear, oxygen and carbon contents in the wear track, and the crystal lattice constant. These parameters are interrelated, and the observed changes in their values are correlated. 

Given the above, movable precision components made of bearing steel (100Cr6) can work in a high ionizing radiation field because their dimensions and friction coefficients will be similar to the designed ones, irrespective of the irradiation fluence (time). This is possible due to fluctuating changes in their crystal lattice constants and tribological properties.

## Figures and Tables

**Table 1:** Content (in wt%) of elements in the surface layer of the analyzed steel grade as measured by EDX.

Sample (Fluence)/Element	Fe	C	Cr	O	Mn	Si	Ni
Unirradiated	93.1 (1)	1.1 (2)	1.5 (2)	0.2 (2)	0.4 (2)	0.3 (2)	0.1 (2)
1.0 × 10^14^ Xe^20+^/cm^2^	93.7 (1)	1.2 (2)	1.6 (2)	0.6 (2)	0.4 (2)	0.3 (2)	0.1 (2)
2.5 × 10^14^ Xe^20+^/cm^2^	93.4 (1)	1.3 (2)	1.6 (2)	0.6 (2)	0.4 (2)	0.3 (2)	0.1 (2)
5.0 × 10^14^ Xe^20+^/cm^2^	92.6 (1)	2.2 (2)	1.5 (2)	1.0 (2)	0.4 (2)	0.3 (2)	0.1 (2)

The numbers in parentheses indicate measurement uncertainties.

**Table 2:** Average content of elements in the wear track on the bearing steel sample surface after 10,000 cycles of tribological testing.

Sample (Fluence)\Element	Fe	C	Cr	O	Mn	Si	Ni
Unirradiated	84.2 (1)	3.9 (2)	1.5 (2)	9.2 (2)	0.3 (2)	0.7 (2)	(2)
1.0 × 10^14^ Xe^20+^/cm^2^	89.2 (1)	3.6 (2)	1.5 (2)	4.8 (2)	0.3 (2)	0.5 (2)	0.1 (2)
2.5 × 10^14^ Xe^20+^/cm^2^	85.7 (1)	4.4 (2)	1.4 (2)	7.4 (2)	0.3 (2)	0.6 (2)	0.1 (2)
5.0 × 10^14^ Xe^20+^/cm^2^	87.3 (1)	3.8 (2)	1.4 (2)	6.3 (2)	0.3 (2)	0.6 (2)	0.1 (2)

The numbers in parentheses indicate measurement uncertainties.

**Table 3:** X-ray beam incidence angle Θ and the calculated X-ray penetration depth (λ = 0.15418 nm) for different reflections (absorption coefficient µ = 2368 cm^−1^).

Incidence Angle Θ (°)	Penetration Depth (µm)		
[hkl]	[011]	[002]	[112]
0.5	0.06 (1)	0.06 (1)	0.06 (1)
1	0.10 (1)	0.10 (1)	0.10 (1)
2	0.19 (1)	0.19 (1)	0.19 (1)
4	0.37 (1)	0.37 (1)	0.37 (1)
7	0.60 (1)	0.62 (1)	0.63 (1)

The numbers in parentheses indicate measurement uncertainties.

**Table 4:** Values of the crystal lattice constant *a* and its changes Δ*a* compared to the unirradiated sample.

Sample (Fluence)	*A*	Δ*a*
Unirradiated	2.8651 (1)	0
1.0 × 10^14^ Xe^20+^/cm^2^	2.8658 (1)	0.0007 (2)
2.5 × 10^14^ Xe^20+^/cm^2^	2.8646 (1)	−0.0005 (2)
5.0 × 10^14^ Xe^20+^/cm^2^	2.8667 (1)	0.0016 (2)

The numbers in parentheses indicate measurement uncertainties.

**Table 5:** Crystal lattice strain *η* and crystallite size *D*.

Fluence/ Incidence Angle Θ (°)	Unirradiated		1 × 10^14^ Xe^20+^/cm^2^		2.5 × 10^14^ Xe^20+^/cm^2^		5 × 10^14^ Xe^20+^/cm^2^	
	η (%)	D (nm)	η (%)	D (nm)	η (%)	D (nm)	η (%)	D (nm)
0.5	0.68	15	0.55	18	0.51	20	0.37	23
1	0.53	16	0.48	18	0.44	20	0.33	23
2	0.50	16	0.43	20	0.40	21	0.27	31
4	0.45	19	0.37	22	0.37	23	0.33	25
7	0.45	19	0.37	27	0.33	25	0.32	26

## Data Availability

The data presented in this study are available on request from the corresponding author.
